# Identification of three podoviruses infecting *Klebsiella* encoding capsule depolymerases that digest specific capsular types

**DOI:** 10.1111/1751-7915.13370

**Published:** 2019-01-31

**Authors:** Yi‐Jiun Pan, Tzu‐Lung Lin, Yi‐Yin Chen, Peng‐Hsuan Lai, Yun‐Ting Tsai, Chun‐Ru Hsu, Pei‐Fang Hsieh, Yi‐Tsung Lin, Jin‐Town Wang

**Affiliations:** ^1^ Department of Microbiology and Immunology School of Medicine China Medical University Taichung Taiwan; ^2^ Department of Medical Biotechnology and Laboratory science College of Medicine Chang Gung University Taoyuan Taiwan; ^3^ Department of Pediatrics College of Medicine Chang Gung Children's Hospital Chang Gung Memorial Hospital Chang Gung University Taoyuan Taiwan; ^4^ Department of Medical Research E‐Da Hospital Kaohsiung Taiwan; ^5^ Department of Microbiology National Taiwan University College of Medicine Taipei Taiwan; ^6^ Division of Infectious Diseases Department of Medicine Taipei Veterans General Hospital Taipei Taiwan; ^7^ Department of Internal Medicine National Taiwan University Hospital Taipei Taiwan

## Abstract

*Klebsiella pneumoniae* is an important human pathogen causing opportunistic nosocomial and community‐acquired infections. A major public health concern regarding *K. pneumoniae* is the increasing incidence of multidrug‐resistant strains. Here, we isolated three novel *Klebsiella* bacteriophages, KN1‐1, KN3‐1 and KN4‐1, which infect KN1, KN3 and K56, and KN4 types respectively. We determined their genome sequences and conducted a comparative analysis that revealed a variable region containing capsule depolymerase‐encoding genes. Recombinant depolymerase proteins were produced, and their enzymatic activity and specificity were evaluated. We identified four capsule depolymerases in these phages that could only digest the capsule types of their respective hosts. Our results demonstrate that the activities of these capsule depolymerases were correlated with the host range of each phage; thus, the capsule depolymerases are host specificity determinants. By generating a capsule mutant, we demonstrate that capsule was essential for phage adsorption and infection. Further, capsule depolymerases can enhance bacterial susceptibility to serum killing. The discovery of these phages and depolymerases lays the foundation for the typing of KN1, KN3, KN4 and K56 *Klebsiella* and could be useful alternative therapeutics for the treatment of *K. pneumoniae* infections.

## Introduction

*Klebsiella pneumoniae* is an important human pathogen that causes opportunistic nosocomial infections, particularly among immunocompromised patients. It is an aetiological agent of septicaemia, pneumonia, soft tissue infections, urinary tract infections, surgical site infections and catheter‐related infections (Podschun and Ullmann, 1998; Abbot, 2003). This pathogen can also cause community‐acquired infections, and over the past three decades, *K. pneumoniae* has become a major cause of pyogenic liver abscess, complicated with metastatic meningitis and endophthalmitis, and this emerging disease has been reported worldwide (Ko *et al*., 2002; Ohmori *et al*., 2002; Okano *et al*., 2002; Chung *et al*., 2008; Tsai *et al*., 2008; Jun, 2018; Wang *et al*., 2018). Another study demonstrated the important role of *K. pneumoniae* in bacteraemic community‐acquired pneumonia (CAP), as this pathogen accounted for more than half of CAP cases in Taiwan (49/95, 51.5%) during 2001–2008 (Lin *et al*., 2010). In several countries, the increasing incidence of drug‐resistant strains of *K. pneumoniae* is a global concern (Wright *et al*., 2014; Pitout *et al*., 2015; Zowawi *et al*., 2015; Mavroidi *et al*., 2016; Rojas *et al*., 2017). Thus, *K. pneumoniae* has been one of the most troublesome Gram‐negative bacteria.

*Klebsiella pneumoniae* is typically coated with a thick capsule layer that has been shown to be crucial for virulence (Fang *et al*., 2004; Chuang *et al*., 2006). Capsule is known to confer resistance to host immune defences (e.g. it protects the bacterium from lethal serum factors and phagocytosis), suppress the early inflammatory response and contribute to biofilm formation (Fang *et al*., 2004; Chuang *et al*., 2006; Llobet *et al*., 2008; Wu *et al*., 2011; Li *et al*., 2014). Furthermore, this polysaccharide coat acts as a physical barrier to the harmful effects of hostile environments, reduces susceptibility to certain antibiotics (Stewart, 1996; Yu *et al*., 2015) and hinders phage access, thus limiting infection (Samson *et al*., 2013). However, capsule may also serve as a receptor for certain phages (Stummeyer *et al*., 2006; Rakhuba *et al*., 2010).

*Klebsiella* capsule structures are highly diverse. From 1926 to 1977, a total of 77 K types were identified by serological reactivity tests (Ørskov and Fife‐Asbury, 1977). In 2008, a new capsular type (KN1) distinct from these 77 types was identified among strains causing pyogenic liver abscess (Pan *et al*., 2008). Subsequently, additional types, including KN2, from a urinary tract infection strain (a KN2‐specific phage and polysaccharide depolymerase were also characterized in the previous study), KN3, from multidrug‐resistant strains, and KN4 and KN5 from bacteraemic isolates, were documented (Hsu *et al*., 2013; Pan *et al*., 2013, 2015b, 2017). The number of novel types is continuously increasing, mainly due to the identification of new capsular polysaccharide (*cps*) gene clusters (Arakawa *et al*., 1995; Shu *et al*., 2009; Fevre *et al*., 2011; Chen *et al*., 2014; Deleo *et al*., 2014; Chung The *et al*., 2015; Wyres *et al*., 2015, 2016), and to date, > 130 capsular types have been identified and certain capsular types are known to be associated with particular diseases (Fung *et al*., 2002; Fang *et al*., 2007), infection severity (Mizuta *et al*., 1983; Cortes *et al*., 2002) or drug resistance (Pan *et al*., 2015b; Huang *et al*., 2018). Capsular types K1 and K2 are considered to be the predominant virulent strains, and their virulence has been confirmed in mouse models (Mizuta *et al*., 1983; Fung *et al*., 2002; Struve *et al*., 2005). Seven capsular types, K1, K2, K5, K20, K54, K57 and KN1, have been reported to be associated with pyogenic liver abscess (Fang *et al*., 2007; Pan *et al*., 2008; Yu *et al*., 2008). Additionally, capsular types K47 and K64 are prevalent in multidrug‐resistant strains (Pan *et al*., 2015b; Huang *et al*., 2018). Notably, due to the limitation of traditional serotyping using antisera, many genotyping methods have been developed to determine the capsular types of clinical isolates. For example, *cps* PCR‐restriction fragment length polymorphism (RFLP) analysis has been used to distinguish the genetic differences among different capsule synthesis clusters; therefore, capsular types can be determined based on their distinct RFLP patterns. Sequencing of *wzi* (that encodes an outer membrane protein involved in capsule attachment to the cell surface) or *wzc* (that encodes a protein kinase involved in the export of capsule) has also been used for capsular typing of *Klebsiella* spp. based on the sequence variation among different types (Brisse *et al*., 2013; Pan *et al*., 2013). PCR‐based genotyping (*cps* PCR genotyping) is another method commonly used to detect unique genes in specific capsular types (Fang *et al*., 2005, 2007; Struve *et al*., 2005; Chuang *et al*., 2006; Yu *et al*., 2007; Pan *et al*., 2008).

Because *K. pneumoniae* is typically surrounded by a polysaccharide‐based capsule, bacteriophages that infect *Klebsiella* may show specificity for various capsular types. Therefore, the use of phages to determine the K types of *Klebsiella* has been described (Rakieten *et al*., 1940; Adams and Park, 1956; Thurow *et al*., 1974; Rieger‐Hug and Stirm, 1981; Gaston *et al*., 1987; Pieroni *et al*., 1994). Moreover, several phages have been reported to carry capsule depolymerases that contribute to the recognition and digestion of capsule; some capsule depolymerases were demonstrated to be critical for phage infection (Hsu *et al*., 2013; Lin *et al*., 2014; Pan *et al*., 2015b, 2017; Hsieh *et al*., 2017). The proteins displaying capsule depolymerase activities are often tail fibres or tail spikes, components of the viral surface that interact with bacterial cells. For these reasons, phage‐borne capsule depolymerases that exhibit high specificity for different capsular types have been suggested for capsular typing. In addition to capsular typing, the therapeutic efficacy of phages and their depolymerases has also been described. A K1 depolymerase was shown to sensitize the *Klebsiella* K1 strain to killing by the complement, and treatment with the phage or the enzyme provided significantly increased survival in mice infected with K1 *Klebsiella* (Lin *et al*., 2014). A K64 depolymerase was demonstrated to enhance serum and neutrophil killing *in vitro* and increase survival rates for K64 *K. pneumoniae*‐inoculated mice (Pan *et al*., 2015b). Treatment with a K5 phage increased the survival of mice infected with a *K. pneumoniae* K5 strain (Hsieh *et al*., 2017). A K63 depolymerase can inhibit *Klebsiella*‐induced mortality of *Galleria mellonella* larvae in a time‐dependent manner (Majkowska‐Skrobek *et al*., 2016). Recently, two additional capsule depolymerases (K3 and K21) were demonstrated to enhance bacterial killing by serum, decrease intracellular survival of phagocytized bacteria in macrophages and increase the lifespan of *Galleria mellonella* larvae infected with *K. pneumoniae*, implicating that capsule depolymerases could be potential agents defeating resistance mechanism of *K. pneumoniae* against the innate immunity (Majkowska‐Skrobek *et al*., 2018).

In the present study, we report three novel *Klebsiella* phages, KN1‐1, KN3‐1 and KN4‐1, infecting KN1, KN3 and K56, and KN4 types respectively. We further identified the specific capsule depolymerases of these phages. The discovery of these phages and depolymerases lays the foundation for typing of KN1, KN3, KN4 and K56 *Klebsiella* and may be used as alternative therapeutics for the treatment of *K. pneumoniae* infections.

## Results

### Isolation of the *Klebsiella* bacteriophages KN1‐1, KN3‐1 and KN4‐1

**Figure 1:**
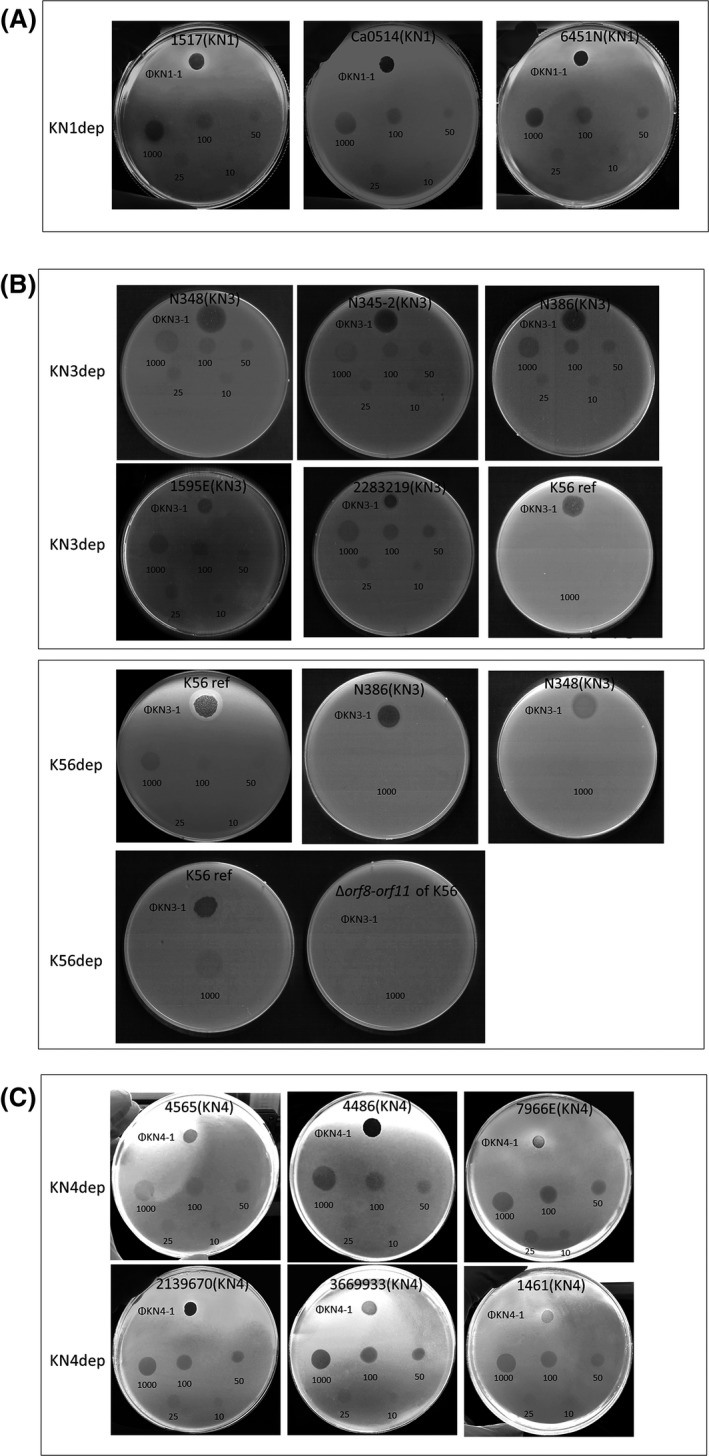
Spot tests of phages KN1‐1, KN3‐1 and KN4‐1 and their capsule depolymerases. Phage (KN1‐1, KN3‐1 or KN4‐1; 10^6^ pfu each) or capsule depolymerase (KN1dep, KN3dep, K56dep or KN4dep; 10–1000 ng) was spotted on the plate. Then, the plates were examined for plaques or semi‐clear zones caused by the phages or depolymerases respectively. Strain name of bacteria was indicated on the top of each agar plate (capsular type was shown in parentheses). Capsule depolymerase was depicted on the left of panels.A. Phage KN1‐1 and KN1dep spotted on KN1 strains.B. Phage KN3‐1 spotted on KN3 strains, a reference K56 strain (abbreviated as K56ref) and the isogenic capsule mutant of K56 (Δ*orf8*‐*orf11* of K56); KN3dep spotted on KN3 strains and a reference K56 strain; K56dep spotted on KN3 strains, a reference K56 strain and the isogenic capsule mutant of K56(Δ*orf8*‐*orf11* of K56).C. Phage KN4‐1 and KN4dep spotted on KN4 strains.

Three *Klebsiella* bacteriophages, KN1‐1, KN3‐1 and KN4‐1, were isolated using *K. pneumoniae* strains A1517, N386 and 4565, which are representative strains of types KN1, KN3 and KN4 respectively. The host ranges of these phages were examined by spotting on lawns of 82 different capsular types of *Klebsiella* (Fig. 1A–C, and data not shown). Additional bacterial strains with the *wzy* genes of types KN1 (6451N and Ca0514), KN3 (N345‐2, N348, 1595E and 2283219) and KN4 (1461, 4486‐2, 7966E, 2139670 and 3669933) were included for sensitivity testing. Phage KN1‐1 formed lytic spots on all three tested KN1‐type strains (A1517, 6451N and Ca0514), but did not cause lytic infections in the other tested types. Similarly, KN4‐1 formed spots on all six KN4 strains (1461, 4565, 4486‐2, 7966E, 2139670 and 3669933), but not on the other types, indicating that the KN1‐1 and KN4‐1 phages are specific to KN1 and KN4 *K. pneumoniae* respectively. In contrast, phage KN3‐1 formed spots not only on the five tested KN3‐type *K. pneumoniae* strains (N386, N345‐2, N348, 1595E and 2283219) but also on the K56 *Klebsiella* reference strain, suggesting that this phage can infect two capsular types, K56 and KN3 (Table S1).

### Morphological characterization of bacteriophages KN1‐1, KN3‐1 and KN4‐1

**Figure 2:**
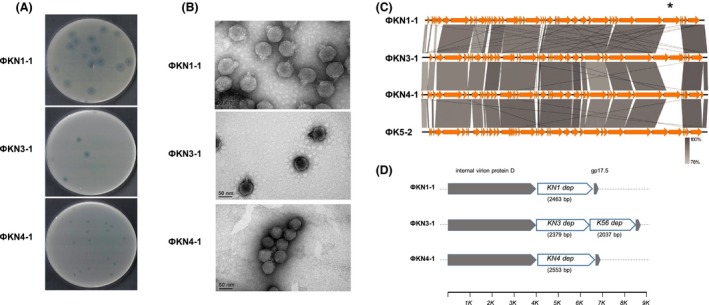
Morphological and molecular characterization of bacteriophages KN1‐1, KN3‐1 and KN4‐1.A. The plaque morphology of phages KN1‐1, KN3‐1 and KN4‐1. Clear plaques surrounded by translucent halos were observed on the individual lawns of *Klebsiella* A1517 (KN1), N386 (KN3) and 4565 (KN4).B. Electron micrographs of phages KN1‐1, KN3‐1 and KN4‐1. Purified phage particles of KN1‐1, KN3‐1 and KN4‐1 exhibited icosahedral capsids and short tails.C. Genome comparisons of phages KN1‐1, KN3‐1, KN4‐1 and K5‐2. Comparative analysis of the phage genomes was performed with Easyfig. The four phage genomes are very similar except for a 2.5–4.5 kb variable region (indicated by an asterisk).D. The variable region. Genes located in this variable region are shown as white arrows, while the conserved genes upstream and downstream are shown in grey. Gene length is shown in parentheses; the axis below shows the position in kb.

Phage KN1‐1 produced 2–3 mm diameter clear plaques surrounded by large halos, and phage KN3‐1 produced 1–2 mm diameter clear plaques surrounded by large halos, whereas phage KN4‐1 produced 1–2 mm diameter clear plaques surrounded by small halos (Fig. 2A). The morphology of the purified phage particles was examined by transmission electron microscopy (TEM; Fig. 2B). The results revealed that these phages had icosahedral capsids and short tails and resembled members of the *Podoviridae*. The average head diameters of KN1‐1, KN3‐1 and KN4‐1 were ~60, 48 and 43 nm, respectively, and the average tail lengths were ~15, 15 and 13 nm respectively (Table S2).

### Analysis of the genome sequences of bacteriophages KN1‐1, KN3‐1 and KN4‐1

Previous studies have been reported that the presence of translucent halos surrounding phage plaques indicates the production of phage‐borne capsule depolymerases (Lin *et al*., 2014); thus, we determined the genome sequences of phage KN1‐1, KN3‐1 and KN4‐1 by high‐throughput sequencing to search for possible capsule depolymerases. The genome of phage KN1‐1 is 40 236 bp in length (Accession No. LC413193), with 52.8% G+C content and 68 probable protein‐coding genes (> 300 bp in length). The genome of phage KN3‐1 is 41 059 bp in length (Accession No. LC413194), with 53.5% G+C content and 60 probable protein‐coding genes (> 300 bp in length). The genome of phage KN4‐1 is 41 219 bp in length (Accession No. LC413195), with 53% G+C content and 67 probable protein‐coding genes (> 300 bp in length). Nucleotide BLAST analysis revealed that the three phages exhibited high DNA similarity with *Klebsiella* phage K5‐2 (Hsieh *et al*., 2017) (Accession No. KY389315.1, 83–85% query coverage and 92–95% identity). *Klebsiella* phage, K5‐2, infects types K5, K30 and K69 and carries K5, K30 and K69 capsule depolymerase‐encoding genes, which are located in a region (between the genes encoding internal virion protein D and gp17.5) that showed low sequence similarity with the corresponding regions of phages KN1‐1, KN3‐1 and KN4‐1 (Fig. 2C and D). Genes from the analogous variable regions in the three currently identified phages were predicted as putative tail fibre/spike encoding genes, suggesting that these proteins may have capsule depolymerization activities (Table 1).

**Table 1:** Capsule depolymerases of *Klebsiella* phages

ORF	Phage	Location	Product size (aa)	NCBI blastp			pfam	
				Homologue (Accession No)	Query cover (%)	Sequence identity	Protein family	Motif, aa
KN1dep	KN1‐1	34320‐36779	820	Tail fibre protein of *Klebsiella* phage JY917 (AVI03134.1)	79	223/686 (33%)	Bacteriophage T7 tail fibre protein	6‐156
KN3dep	KN3‐1	33247‐35622	792	Tail fibre protein of *Klebsiella* phage K5‐2(APZ82804.1)	39	256/311 (82%)	Bacteriophage T7 tail fibre protein	3‐154
							Pectate lyase superfamily protein	300‐514
KN4dep	KN4‐1	34859‐37408	850	Tail fibre protein of *Klebsiella* phage 2044‐307w(ASZ78307.1)	18	124/154 (81%)	Bacteriophage T7 tail fibre protein	4‐156
K56dep	KN3‐1	35635‐37668	678	Tail fibre protein of *Klebsiella* virus vB_KpnM_KpS110(AUV59230.1)	94	158/672 (24%)	N.A.^a^	N.A.^a^

a None predicted.

### Expression of the putative capsule depolymerases

We cloned and expressed the *KN1dep* and *KN4dep* genes from phages KN1‐1 and KN4‐1, respectively, as well as two genes, *KN3dep* and *K56dep*, from phage KN3‐1 via a pET‐28c expression system. To determine whether these putative tail fibres/spikes had capsule‐digesting activities, the resulting purified proteins were assessed by spot tests against their hosts: KN1‐type *K. pneumoniae* A1517, 6451N and Ca0514 for phage KN1‐1; KN3‐type strains N386, N345‐2, N348, 1595E, 2283219 and a K56 *Klebsiella* reference strain for phage KN3‐1; and KN4 strains 1461, 4565, 4486‐2, 7966E, 2139670 and 3669933 for phage KN4‐1. The results revealed that the putative tail fibres/spikes generated semi‐clear spots on lawns of their respective hosts. Specifically, KN1dep exhibited capsule depolymerization activities against all three KN1‐type *K. pneumoniae* (Fig. 1A), KN3dep exhibited activities against all five KN3‐type strains but not the K56 strain (Fig. 1B), K56dep showed activity against the K56 strain but not the KN3 strains (Fig. 1B), and KN4dep showed activity against all six KN4 strains (Fig. 1C). The specificities of these capsule depolymerases were further clarified by testing against all other documented capsular types, including 77 references strains and KN1–KN5 strains (Pan *et al*., 2008, 2013, 2015b; Hsu *et al*., 2013) (data not shown). These results revealed that each of the four enzymes could only digest capsule from the respective unique capsular type (as indicated above), indicating the specificity of these enzymes. When different amounts of capsule depolymerases were tested, translucent spots were formed with 10–1000 ng of KN1dep, KN3dep and KN4dep (Fig. 1A–C), while relatively weak translucent spots were formed with ≤ 50 ng of K56dep (Fig. 1B).

### Alcian blue staining of enzyme‐digested capsular polysaccharide (CPS)

**Figure 3:**
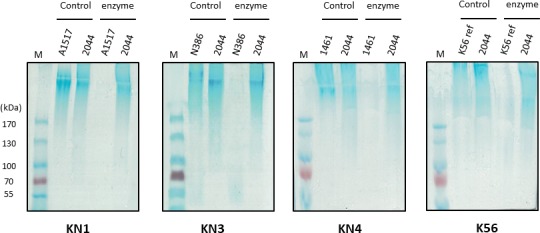
Alcian blue staining of CPS treated with the KN1dep, KN3dep, KN4dep and K56dep capsule depolymerases. Extracellular polysaccharides were extracted from A1517 (KN1 type), N386 (KN3 type), 1461 (KN4 type) and a reference K56 strain treated with or without 5 μg of KN1dep, KN3dep, KN4dep and K56dep enzyme, respectively, and then visualized by Alcian blue staining. In each panel, lane 1 (M) is a protein marker; lanes 2 and 3 are controls without enzyme; and lanes 4 and 5 are enzyme‐treated. An irrelevant capsular‐type strain, NTUH‐K2044 (K1), abbreviated as 2044, was used as a control. K56 reference strain is abbreviated as K56ref.

The capsule‐degrading activities of KN1dep, KN3dep, KN4dep and K56dep were further validated by Alcian blue staining of enzyme‐treated CPS (Fig. 3). The results revealed that untreated CPS was present as higher molecular weight capsular polysaccharide polymers at the top of an SDS‐PAGE gel. In contrast, when KN1, KN3, KN4 and K56 CPS were treated with KN1dep, KN3dep, KN4dep and K56dep (5 μg, each), respectively, lower molecular weight material was observed. A strain of an irrelevant capsular type, NTUH‐K2044 (K1), was used as a control, and its insusceptibility to KN1dep, KN3dep, KN4dep and K56dep indicates the high specificity of these capsule depolymerases.

### Detection of reducing sugars from enzyme‐digested capsular polysaccharide

**Figure 4:**
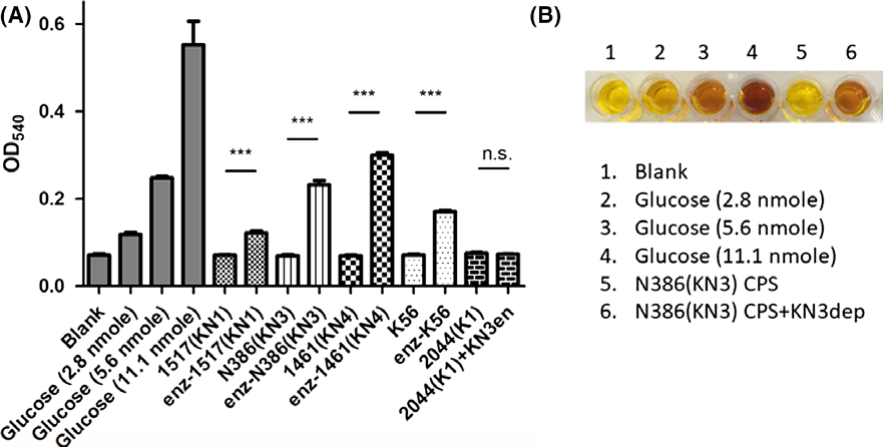
Enzymatic activity displayed by capsule depolymerases.A. 10 μg CPS was incubated with 1 μg capsule depolymerase at 37°C for 1 h and then was reacted with dinitrosalicylic acid (DNSA). The absorbance at 540 nm was determined in extracted CPS with or without enzyme treatment. Enzyme‐treated groups (CPS was treated with respective enzyme) were shown as enz‐. Different amounts of glucose were used as controls. ***P < 0.001; n.s., not significant.B. Colour change in DNSA reacted with KN3 CPS. NTUH‐K2044 is abbreviated as 2044.

In order to confirm that the capsule is the target of depolymerase, the degradation of CPS was further assayed with dinitrosalicylic acid (DNSA) by detecting the release of reducing ends after incubated with the depolymerase. If the enzyme can digest CPS, additional reducing ends would be generated as the polysaccharides were cleaved into small subunits. DNSA was reduced while oxidizing the reducing sugar, and colour change was detected at a wavelength of 540 nm. The results indicated that KN1dep, KN3dep, KN4dep and K56dep can degrade the respective CPS, as evidenced by an increase in concentration of reducing ends after enzyme treatment. In contrast, no increase in reducing sugar was observed when enzyme incubated with irrelevant type of CPS (K1 CPS was not digested by KN3dep), which proves the specificity of the capsule depolymerase (Fig. 4A and B).

### Infectivity of the phage towards different strains

We also compared the phage infectivity and capsule depolymerase activity in different host strains. An efficiency‐of‐plating (EOP) assay was used to quantitate the ability of phage to infect different hosts (Fig. S1). The results indicated that phage KN1‐1 has similar infectivity towards all three tested KN1 hosts (no significant difference was observed between A1517 and Ca0514, or A1517 and 6451N), whereas phage KN3‐1 showed lower infectivity towards 1595E (~3% compared with that of N348, which was set at 100%). Different levels of infectivity were also observed for phage KN4‐1 and the KN4 hosts. The EOP value indicated that the infectivity of 2139670 by KN4‐1 was about threefold higher than that of 4565, and the infectivity of 7966E and 3669933 was relatively low (0.07% and 0.9% respectively). Interestingly, no plaques were observed on 1461 even though phage KN4‐1 formed spots on 1461 by spot test. To determine whether phage KN4‐1 formed small plaques which is barely visible on 1461 or 1461 is resistant to infection of phage KN4‐1, a total of 5 × 10^3^ pfu phage was co‐incubated with 5 × 10^6^ cfu of 1461 or 4565 and phage titre was determined at 0.5, 1, 3 and 22 h by plating on 4565 strain. The results indicated that phage number continuously increased when incubated phage KN4‐1 with 4565 but not with 1461 (even though phage KN4‐1 can adsorb to 1461 bacterial cells) (Fig. S2A and B). After 22 h of incubation with 4565, phage number reached ~10^9^–10^10^ pfu/ml, and the bacterial culture appeared clear (lysis); in contrast, bacterial culture of 1461 showed turbid (growth) after incubation (Fig. S2C). Taken together, it suggested that phage KN4‐1 may only make a halo zone by depolymerase when spotting on 1461 strain, but was unable to successfully infect 1461 strain to produce progeny viruses and kill the host. Although the infectivity of the phages differed among strains with same capsular types, aliquots containing 10 ng or more of recombinant KN1dep, KN3dep and KN4dep consistently formed translucent spots on all tested hosts (Fig. 1A–C).

### Infectivity of the phage KN3‐1 to a capsule mutant of K56 strain

To clarify whether the bacterial capsule was essential for infection by the phages, an isogenic capsule‐deficient mutant of K56, in which *orf8*‐*orf11* (predicted to encode glycosyltransferases) of the capsular polysaccharide biosynthesis loci were deleted, and the capsule loss in Δ*orf8*‐*orf11* of K56 was confirmed by Alcian blue staining (Fig. S3). Spot tests showed that Δ*orf8*‐*orf11* of K56 was not infected by KN3‐1 phage, in contrast to the parental strain (Fig. 1B), and K56dep protein can only cause semi‐clear spots on wild‐type K56 but not on Δ*orf8*‐*orf11* of K56 (Fig. 1B). These results indicated that capsule was the target of depolymerase and essential for infection by this phage, implying that the capsule might be the receptor required for phage infection.

### Adsorption of phage KN3‐1 to wild‐type K56 or capsule mutant of K56

We further examined whether the adsorption of phage KN3‐1 to K56 strain was affected by the lack of capsule. Adsorption assay was performed by preincubating phage KN3‐1 with wild‐type K56, Δ*orf8*‐*orf11* of K56 and 4565(KN4). The results indicated that preincubation of ~ 1 × 10^6^ pfu phage KN3‐1 with K56 strain for 5 min resulted in ~500‐fold decrease in virions (the remaining phages were about 2 × 10^3^ pfu), whereas no significant loss of phage particle was observed when phage KN3‐1 preincubated with Δ*orf8*‐*orf11* of K56 or a non‐KN3‐1 host (4565‐ KN4 strain), suggesting that capsule is crucial for attachment of phages to bacterial cells (Fig. S4).

### Serum sensitivity of enzyme‐treated bacteria

**Figure 5:**
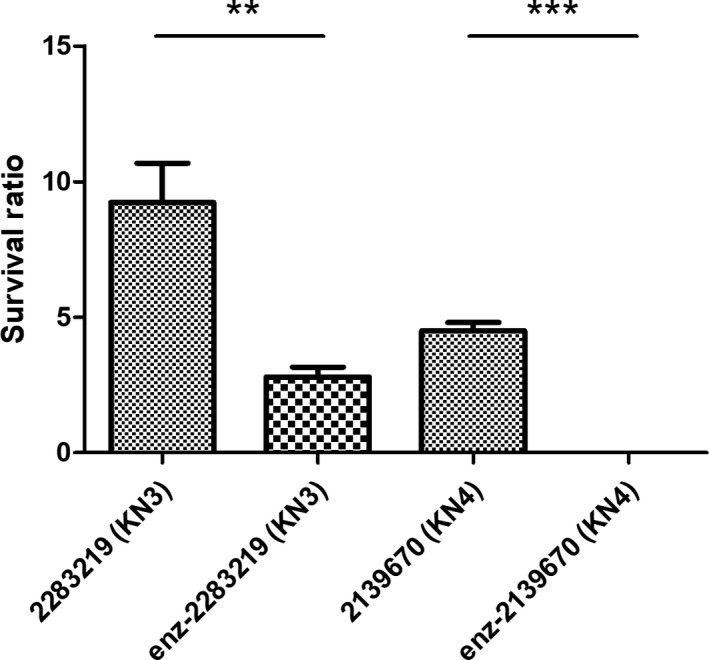
The survival of enzyme‐treated bacteria by serum killing. Bacterial susceptibility to killing by human serum of two serum‐resistant strains, 2283219(KN3) and 2139670(KN4), with or without enzyme pretreatment was presented. Survival ratio indicates per cent survival of bacteria after 3 h of incubation with serum (viable counts relative to initial inoculum). Three independent experiments were performed, and the survival rates of the two groups, control and enzyme pretreated groups (shown as enz‐), were compared by Student's *t*‐test analysis. **P < 0.01; ***P < 0.001.

The effects of serum killing with or without pretreatment of capsule depolymerases for two serum‐resistant strains, 2283219(KN3) and 2139670(KN4), were assessed. Compared to the control (without pretreatment of KN3dep enzyme) which can proliferate with 75% serum (~10‐fold increase in bacterial load), bacterial load of KN3dep‐treated 2283219(KN3) was significantly decreased after incubated with serum for 3 h (*P* = 0.0021, Student's *t*‐test); for 2139670(KN4), the KN4dep‐treated bacteria became sensitive to serum killing (Fig. 5) (*P *= 0.0001, Student's *t*‐test). Thus, our results indicated that capsule depolymerases can enhance bacterial susceptibility to serum killing.

## Discussion

Serological typing is the traditional way of detecting the capsule phenotype of *Klebsiella*. However, this method is notoriously difficult because of the limited assay sensitivity and specificity (due to frequent serological cross‐reactivity between different capsular types), high cost, limited sources of antisera and tedious experimental procedures. For this reason, capsular genotyping approaches that do not require the use of antisera, such as *cps* PCR‐RFLP (Brisse *et al*., 2004), *wzi* or *wzc* sequencing (Brisse *et al*., 2013; Pan *et al*., 2013), and *cps* PCR genotyping, have been developed for the determination of capsular types in *Klebsiella* (Fang *et al*., 2005, 2007; Struve *et al*., 2005; Chuang *et al*., 2006; Yu *et al*., 2007; Pan *et al*., 2008). However, these methods have limitations. For example, *cps* PCR‐RFLP requires PCR amplification of the *cps* gene cluster (~20 kb), which can be very difficult in some strains due to sequence variations. In addition, *cps* PCR genotyping can only be used for capsular types with *cps* genes of known sequence, and a separate pair of primers is needed for each type. Although *wzi* or *wzc* sequencing can be used to identify both previously reported and novel types, one‐run sequencing of PCR products is needed. Most importantly, these genotyping‐based strategies detect the capsular genotype but not the capsular phenotype. Genotype analysis, particularly analysis based on a partial sequence of *cps*, could miss subtle changes in sugar structures that may arise from small‐scale insertions/deletions and point mutations. For example, the K22 and K37 capsules have indistinguishable *cps* regions but differ in the presence/absence of an acetyl group modification (Parolis *et al*., 1988). The difference arises from a sequence difference (a frameshift mutation resulting from a single nucleotide deletion) in a putative acetyltransferase. Thus, these two capsular types cannot be differentiated by *cps* PCR‐RFLP, *wzi*/*wzc* sequencing or *cps* PCR genotyping. In addition, non‐capsulated mutants that have defects in capsule expression would not be accurately characterized. This could be avoided by phage/depolymerase typing, which is possibly a rapid and easy method for detecting the expressed capsular type.

Our present work documented three phages infecting *Klebsiella*. Despite that all the three phages can form plaques surrounded by translucent halos, the size of clear plaques and halos is different among the three phages. According to a previous study which demonstrated that the size of clear plaque was associated with various properties of phage, such as adsorption rate, burst size, lysis time, virion size and diffusivity (Gallet *et al*., 2011), we speculated that these factors may contribute to the difference of plaque formation in our phages. As the halo is caused by phage‐borne depolymerases, it suggested that the enzymatic activities and amount of enzyme production could affect the size of halos. Host range determination indicated that one phage each is specific for KN1 and KN4 and the remaining one infects both KN3 and K56. We analysed the infectivity of these phages in strains with the same capsular types and tested the sensitivity of these strains to recombinant phage‐encoded capsule depolymerases. The results indicated that while phage infectivity varied, depolymerase activity was consistent among different strains of the same capsular types. In particular, our data suggested that 1461 could be a KN4 strain‐resistant to phage KN4‐1 because the phage failed to propagate with 1461 strain. Alcian blue staining demonstrated that the capsule of 1461 can be digested by KN4dep, and spot test showed that enzyme activity against 1461 is similar to that against other KN4 strains. Thus, applications using capsule depolymerases for capsular typing would likely yield more consistent results than applications using phages. In addition, capsule depolymerases have higher specificity than phages because some phages can infect more than one capsular type (Pan *et al*., 2015b). Likewise, our currently identified KN3‐1 phage infects both KN3 and K56 and carries two specific capsule depolymerases, KN3dep and K56dep, which are specific for KN3 and K56 type respectively. Therefore, capsule depolymerases could show higher specificity than phages for capsular typing.

The high specificity of capsule depolymerase suggested that capsule depolymerase could recognize specific sugar linkage of different capsule, and thus, we speculated that polysaccharides are likely digested into oligosaccharides other than monosaccharides. This notion was evidenced by previous studies, which reported that the cleavage of capsular polysaccharides with phage‐borne polysaccharide depolymerases gave oligosaccharide products. For example, a mixture of octasaccharides through disaccharides were proved to be the depolymerization products of K5 *Escherichia coli* capsule reacted with phage‐encoded capsule depolymerase(O'Leary *et al*., 2013); hexasaccharides were the primary degradation product of K12 *E. coli* capsule cleaved by phage‐associated depolymerase(Altmann *et al*., 1990); octasaccharides were the main product when the capsule of *Acetobacter methanolicus* was fragmented by phage‐associated depolymerase (Grimmecke *et al*., 1991).

To our knowledge, 19 capsule depolymerases have been identified (capsule‐degrading activity has been demonstrated and the sequence is available) for 17 *Klebsiella* capsular types (Table S4), including three K1 enzymes (Lin *et al*., 2014; Pan *et al*., 2017; Solovieva *et al*., 2018), one K2/K13 enzyme (Solovieva *et al*., 2018), one K3 enzyme (Majkowska‐Skrobek *et al*., 2018), one K5 enzyme (Hsieh *et al*., 2017), one K8 enzyme (Hsieh *et al*., 2017), two K30/K69 enzymes (Hsieh *et al*., 2017; Pan *et al*., 2017), one K11 enzyme (Pan *et al*., 2017), two K21 enzymes (Pan *et al*., 2017) (Majkowska‐Skrobek *et al*., 2018), one K25 enzyme (Pan *et al*., 2017), one K35 enzyme (Pan *et al*., 2017), one K63 enzyme (Majkowska‐Skrobek *et al*., 2016), one K64 enzyme (Pan *et al*., 2017), one KN2 enzyme (Hsu *et al*., 2013), one KN4 enzyme (Pan *et al*., 2017) and one KN5 enzyme (Pan *et al*., 2017). We analysed the sequence similarity of enzymes that depolymerize the same capsular type. Two proteins from two highly related K1‐infecting podoviruses, K1‐ORF34 from phage NTUH‐K2044‐K1‐1 and kpv71_52 from phage vB KpnP KpV71, showed high amino acid sequence conservation (100% query coverage and 98% identity), and the genomes of these phages also have high similarity (91% query coverage and 95% identity), indicating that these two phages may be derived from the same lineage. However, the other identified K1 enzyme, S2‐4, from the myovirus K64‐1 exhibited limited sequence similarity with K1‐ORF34 (77% query coverage and 33% identity), indicating that there are (at least) two variant K1 depolymerases from phylogenetically distinct phages. The two K30/K69 enzymes, S2‐6 from phage K64‐1 and ORF37 from phage K5‐2 (Hsieh *et al*., 2017; Pan *et al*., 2017), exhibited limited amino acid sequence identity (73% query coverage and 50% identity); the two K21 enzymes, S1‐3 from phage K64‐1 and KP32gp38 from phage KP32 (Majkowska‐Skrobek *et al*., 2018), exhibited limited amino acid sequence identity (88% query coverage and 39% identity). Comparison of one of the currently isolated proteins, KN4dep, to S1‐2 protein from phage K64‐1 also showed limited amino acid sequence identity (14% query coverage and 50% identity). The properties of enzymes that depolymerize the same capsular type should be further analysed and compared to help choose the best candidates for future applications.

The genomes of the three podoviruses identified in this study showed high levels of sequence similarity, except for one region with considerable sequence variation. The genes located in this region have capsule depolymerase activities. These results were consistent with a previous study, which showed that this analogous variable region (between the genes encoding internal virion protein D and gp17.5) harbours genes encoding capsule depolymerases in different T7‐like phages (Hsieh *et al*., 2017). Thus, PCR amplification of this region from other phages belonging to this group will be useful for the identification of capsule depolymerases (Hsieh *et al*., 2017).

Similar to previous observations (Lin *et al*., 2014; Hsieh *et al*., 2017), we demonstrated that capsule mutants became resistant to phage infection, suggesting that the capsule was essential for phage infection. In addition, adsorption experiments indicated that the capsule might be a receptor crucial for attachment of phages to bacterial cells. These results are consistent with that proteins exhibited capsule depolymerase activities were predicted as putative tail fibres/spikes which may contact with the surface of bacteria and are responsible for host cell recognition.

In the present study, we identified three *Klebsiella* bacteriophages and characterized their capsule depolymerases. The capsule depolymerase activities were correlated with the host range of each phage, i.e. phage KN1‐1, which is specific for KN1‐type *Klebsiella*, encodes a KN1 capsule depolymerase; phage KN4‐1, which is specific for KN4‐type *Klebsiella*, encodes a KN4 capsule depolymerase; and phage KN3‐1, which infects KN3‐ and K56‐type *Klebsiella*, encodes a KN3 capsule depolymerase and a K56 depolymerase. Thus, the capsule depolymerases are host specificity determinants of these phages. Further, the K56, KN1, KN3 and KN4 capsule depolymerases can only digest capsule from the respective unique capsular type, indicating that the structures of the KN1, KN3 and KN4 capsules are distinct from other reported capsular types. Because of their high‐level specificity and sensitivity, these capsule depolymerases may be useful for capsular typing in epidemiological investigations. We determined the sequences of these capsule depolymerases and demonstrated their *in vitro* activity, and this information will be useful for investigating the properties of these depolymerases, such as their binding and catalytic domains, the enzymatic reactions and the interactions between CPS and enzyme. In addition, the serum killing experiments indicated capsule depolymerase can efficiently reduce the bacterial resistance against serum. Therefore, these newly identified phages and capsule depolymerases could be used as alternative therapeutics for the treatment of multidrug‐resistant *K. pneumoniae* infections in the future (particularly for the KN3 type, which was previously identified among multidrug‐resistant strains and accounts for 4% of the drug‐resistant isolates (Pan *et al*., 2015b)).

## Conclusions

Due to the limitations of traditional serotyping with antisera, new strategies for capsular typing of *Klebsiella* have been developed. One of these approaches is phage/depolymerase typing. Here, we identified three novel phages, determined their host ranges, sequenced their genomes and characterized four capsule depolymerases that are specific for four different capsular types. These results provide evidence for the association between the molecular characteristics of phages/phage‐encoded capsule depolymerases and host specificity as well as important information for capsular typing applications. In addition, these phages/depolymerases could be used for alternative non‐antibiotic treatment of infections in the future.

## Experimental procedures

### Bacterial strains

Strains representing 82 capsular types were used for host range determination, including 77 *Klebsiella* reference strains purchased from Statens Serum Institut (Copenhagen, Denmark); four previously reported ‘new’ type strains (A1517 [KN1], Ca0507 [KN2], N386‐KCR59 [KN3], 1461 [KN4] and ca0431 [KN5]) (Pan *et al*., 2008, 2013, 2015b; Hsu *et al*., 2013); and additional KN1 (6451N and Ca0514), KN3 (N345‐2‐KCR57, N348‐KCR58, 1595E and 2283219) and KN4 (4565, 4486‐2, 7966E, 2139670 and 3669933) strains (Table S1; Pan *et al*., 2013, 2015b).

### Phage isolation

Phages that infect KN1, KN3 and KN4 *K. pneumoniae* strains were isolated from the same sewage sample collected at Taipei City and were named KN1‐1, KN3‐1 and KN4‐1 (Hsu *et al*., 2013). Briefly, *K. pneumoniae* was co‐cultured with untreated water in Luria–Bertani (LB) broth (Bioshop, Burlington, Canada) overnight. After centrifugation, the supernatant was filtered through a 0.45 μm filter (Merck Millipore, Tullagreen, Germany) and spotted on LB plates overlaid with *K. pneumoniae* to detect phage plaques. The agar overlay method was used to isolate a pure phage preparation and for titre determination (Pieroni, 1996).

### Efficiency‐of‐plating (EOP) assay

An efficiency‐of‐plating (EOP) assay was used to quantitate the ability of phage to infect different hosts as previously described (Pieroni *et al*., 1994). The EOP value is a ratio indicating how well a bacteriophage forms plaques on different strains compared with the host from which the phage preparation was made. Briefly, samples of serial 10‐fold dilutions of the respective phage suspensions (final concentration 1 × 10^1^ ~ 1 × 10^9^/ml) were prepared with SM buffer, containing 100 mM NaCl, 8 mM MgSO_4_·7H_2_O and 50 mM Tris (pH 7.5) (Sigma‐Aldrich, St Louis, MO, USA). For plating, 100 μl of exponential phase culture of tested strain (1 × 10^8^ cfu/ml) was mixed with 100 μl of phage suspension and incubated for 10 min, and then, 4 ml 0.7% top agar was added. The mixture was lightly vortexed, layered on a LB agar, incubated at 37°C for 18 h and scored for the presence of plaques. The lytic activity of phage on the host bacterial strain used for original titre determination was set to 100 (%) (i.e. A1517, N348 and 4565 for phage KN1‐1, KN3‐1 and KN4‐1 respectively). For example, the ratio of Ca0514 was calculated by the phage titre on Ca0514 compared with the titre on A1517. Due to the large range of data, the logarithmic scales, EOP (Log_10_), are shown. The values of each strains were compared with the value of the host, which was set to 100 (Log_10_ = 2) by paired *t*‐test from three independent experiments.

### Determination of host range and capsule depolymerase activity

Spot tests (Verma *et al*., 2009) were performed to determine whether a bacterial strain is permissive for phage infection; the assay was also used to verify the activity of the capsule depolymerases. Briefly, a 9 cm diameter LB agar plate was overlaid with top agar mixed with 200 μl of a fresh bacterial culture. After the top agar had solidified, aliquots of phage (10^6^ pfu) or various amounts of purified recombinant capsule depolymerase were spotted on the plate. After overnight incubation at 37°C, lytic or semi‐clear spots were observed.

### Transmission electron microscopy (TEM)

Phages were purified by CsCl density gradient centrifugation. Briefly, the phage suspension was layered on the top of a CsCl step gradient (densities: 1.1 g/ml and 1.7 g/ml) (Sigma‐Aldrich, St Louis, MO) and centrifuged in a SW41 Ti swinging bucket rotor at 66 000 × *g* for 16 h at 4°C. After ultracentrifugation, phages were collected from the visible hazy blue/white bands using a syringe with a 23 G needle, and the majority of the CsCl was removed by buffer exchange in ddH_2_O using an Amicon Ultra centrifugal filter (100 000 MWCO; Millipore). Purified phage samples were applied to carbon‐coated nitrocellulose grids, subjected to negative staining with 2% uranyl acetate and examined with a Hitachi H‐7100 transmission electron microscope.

### Genome sequence analysis

Phage genomic DNA was extracted by a Qiagen Lambda Kit (Qiagen, Valencia, CA). After phages were precipitated and lysed, the phage DNA was extracted by phenol/chloroform and then precipitated by ethanol. Genomic sequencing was performed using the Illumina/Solexa GAII sequencing platform by the High‐throughput Genome Analysis Core at Yang‐Ming Genome Research Center with the processing and assembly methods described below. Fifty nanograms of DNA was subjected to construct sequencing library using Illumina‐compatible Nextera DNA Sample Prep Kit (DNA Sample Preparation Kit (Epicentre, USA)) according to the manufacturer's instruction. The constructed library was quantified by quantitative PCR, and the library size was determined on a 2100 Bioanalyzer (Agilent, USA) with High Sensitivity DNA Chip. Sequencing was done on a HiSeq 2000 (Illumina) by paired‐end sequencing with 100 bp read length at the Genome Research Center of National Yang‐Ming University. The sequencing reads were trimmed for a quality lower than Q20 and adapters, followed by *de novo* assembling with CLC Genomics Workbench (CLC bio, Denmark). Gaps were further filled by Sanger sequencing. Coding sequences were predicted by Vector NTI and annotated by NCBI protein BLAST. We also re‐examined the sequences of genes encoding capsule depolymerases by Sanger sequencing (see supplementary materials) and confirmed that the sequences are identical to those from high‐throughput sequencing. Comparative genomic analysis of the phages and visualization of the coding regions were performed with Easyfig (Sullivan *et al*., 2011).

### Expression and purification of putative capsule depolymerases

To generate N‐terminal (His)_6_‐tagged capsule depolymerase proteins, the ORFs (including stop codons) were inserted into the pET‐28c expression vector (Novagen, Madison, WI, USA) via the NheI and XhoI (*KN3dep*), SacI and XhoI (*K56dep*), BamHI and SacI (*KN4dep*), or NotI (*KN1dep*) restriction sites. Primers used for construction of the capsule depolymerase expression vectors are listed in Table S2. PCR amplification was performed with the Long and Accurate PCR system (Takara, Tokyo, Japan). The cycling program was 96°C for 3 min, followed by 30 cycles of 96°C for 30 s, 50°C for 15 s and 72°C for 3 min. The PCR products were ligated into the expression vector after restriction enzyme digestion. The resulting plasmids were transformed into *Escherichia coli* BL21(DE3) cells, and expression was induced by the addition of 0.1 mM IPTG and incubation at 15°C overnight. The resulting His‐tagged proteins were purified using nickel beads (GE Healthcare, Uppsala, Sweden) according to the manufacturer's instructions.

### Extraction and quantification of CPS

The bacterial CPS was extracted by using a method described by Domenico (Domenico *et al*., 1989). Briefly, 500 μl of overnight‐cultured bacterium was mixed with 100 μl of 1% Zwittergent 3–14 (Sigma‐Aldrich, Milwaukee, WI, USA) in 100 mM citric acid (pH 2.0) and then incubated at 50°C for 20 min. After centrifugation, 250 μl of the supernatant was transferred to a new tube, and the CPS was precipitated with 1 ml of ethanol. The mixture was incubated at 4°C for 20 min. After centrifugation, the pellet was dried and dissolved in 100 μl of distilled water. The sample was appropriately diluted, and then, 1200 μl of 12.5 mM sodium tetraborate (Sigma‐Aldrich, St Louis, MO) in H_2_SO_4_ was added. The mixture was vigorously mixed and boiled for 5 min. After cooling, 20 μl of 0.15% 3‐hydroxydiphenol (Sigma‐Aldrich, Milwaukee, WI) was added. The tubes were shaken, and the absorbance at 520 nm was measured. Uronic acid content was determined from a standard curve of d‐glucuronic acid (Sigma‐Aldrich, Milwaukee, WI).

### Alcian blue staining

A total of 2.5 μg of extracted samples were treated with 5 μg capsule depolymerase for 3 h and separated by 10% sodium dodecyl sulfate–polyacrylamide gel electrophoresis (SDS‐PAGE), and capsule was detected with Alcian blue (Sigma‐Aldrich, St Louis, MO) as previously described (Moller *et al*., 1993; Zamze *et al*., 2002). In brief, after electrophoresis, the gel was washed three times (for 5, 10 and 15 min) with fix/wash solution (25% ethanol and 10% acetic acid in water) at 50°C. Then, the gel was soaked in 0.125% Alcian blue dissolved in fix/wash solution (for 15 min in the dark at 50°C) and finally destained with fix/wash solution (overnight at room temperature). CPS was visualized as blue‐stained material.

### Determination of reducing sugars

The dinitrosalicylic acid (DNSA) colorimetric method (Miller, 1959) was used to detect the reducing sugars released by degradation of CPS by capsule depolymerases. A total of 10 μg CPS was incubated with 1 μg capsule depolymerase at 37°C for 1 h and then was reacted with equal volume of the modified DNSA reagent containing 1% DNSA, 0.2% phenol, 0.05% sodium sulfite and 1% sodium hydroxide (Sigma‐Aldrich, St Louis, MO). The mixtures were heated for 5 min in a boiling water bath and then cooled under running tap water adjusted to ambient temperature. The absorbance at 540 nm was determined by Thermo Scientific Multiskan GO spectrophotometer. Different amounts of glucose were used as controls. Three independent experiments were performed, and Student's *t*‐test analysis was conducted.

### Construction of a capsule mutant of the K56 strain

The capsular polysaccharide biosynthesis gene cluster (Accession No. AB924593) of the *Klebsiella* K56 reference strain was published, and *orf8*‐*orf11* were predicted to encode glycosyltransferases, which are considered to be essential for capsule biosynthesis (Pan *et al*., 2015a). To generate a Δ*orf8*‐*orf11* mutant of the K56 reference strain, a previously described unmarked deletion method by use of pKO3‐Km plasmid was applied (Hsieh *et al*., 2008). The flanking regions of *orf8*‐*orf11* were amplified with specific primers (Table S2) and cloned into the pKO3‐Km plasmid. Electroporation, temperature shifts and PCR were performed to select mutants.

### Phage adsorption assay

A phage preparation of 1 × 10^6^ pfu was made for phage KN3‐1, and the titre was determined using K56 strain. Adsorption assay (Scholl *et al*., 2001) was performed by preincubating phage KN3‐1 with 1 × 10^9^ cfu of K56, Δ*orf8*‐*orf11* of K56 and 4565 (the KN4 strain was used as a control) in a total of 1 ml individually. The multiplicity of infection (MOI) was 1 phage particle to 1000 bacterial cells. After preincubation for 5 min at room temperature, the mixture was immediately filtered by 0.45 μm pore size hydrophilic polyethersulfone (PES) membrane (Merck Millipore, Tullagreen, Germany). Phage particles that had attached to bacteria will be eliminated from the filtrate (the remaining would be unadsorbed phages). The filtrate was then titred on K56. Three independent experiments were performed, and Student's *t*‐test analysis was conducted. Similar experiment was performed for phage KN4‐1 by use of bacterial strains 4565, 1461 and K56 (as a control).

### Phage amplification with *Klebsiella strains* 4565 and 1461

A total of 5 × 10^3^ pfu of phage KN4‐1 was mixed with 5 × 10^6^ cfu of exponential phase culture of 1461 or 4565 strain individually. The culture was continuously incubated at 37°C. Samples were taken at 0.5, 1, 3 and 22 h. After adding CHCl_3_ (Sigma‐Aldrich, St Louis, MO) to a final concentration of 2% and shaking for 5 min (treated with CHCl_3_, both intracellular and free phages were determined), the culture was appropriately diluted (or undiluted) with SM buffer, containing 100 mM NaCl, 8 mM MgSO_4_·7H_2_O and 50 mM Tris (pH 7.5) (Sigma‐Aldrich, St Louis, MO). For plating, 100 μl of phage suspension was mixed with 100 μl of exponential phase culture of 4565 strain (we use 4565 but not 1461 because phage numbers can be estimated by calculating the visible plaques on 4565). After incubation at 37°C for 10 min, 4 ml 0.7% top agar was added. The mixture was lightly vortexed, poured on a LB agar and incubated at 37°C for 18 h. Phage titres at each time point were determined by plaque counting.

### Serum killing assay

The serum killing assay of *K. pneumoniae* was performed as previously described (Fang *et al*., 2004). A total of 10^6^ cfu of bacteria were pretreated with or without capsule depolymerase with a final concentration of 150 μg/ml at 37°C for 1 h. Then, an inoculum of 2.5 × 10^4^ cfu of bacteria was mixed with human serum from healthy volunteers at a 1:3 volume ratio. The mixture was incubated at 37°C for 3 h. Per cent survival of bacteria with or without pretreatment of capsule depolymerase was calculated based on viable counts relative to initial inoculum. Three independent experiments were performed, and the survival rates of the two groups, control and enzyme pretreated groups, were compared by Student's *t*‐test analysis.

### Nucleotide sequence accession numbers

The genome sequences of the *Klebsiella* phages KN1‐1, KN3‐1 and KN4‐1 were deposited into GenBank under Accession Nos. LC413193, LC413194 and LC413195 respectively.

## Conflict of interest

The authors declare that they have no competing financial interests.

## Supporting information


**Fig. S1.** Infectivity of phages KN1‐1, KN3‐1, and KN4‐1 toward different strains.
**Fig. S2.** Co‐incubation of phage KN4‐1 with 1461 or 4565.
**Fig. S3.** Alcian blue staining of CPS from K56 reference strain and Δ*orf8‐orf11* of K56.
**Fig. S4.** Adsorption experiments of phage KN3‐1.
**Table S1.** *Klebsiella* strains used in this study and the host range of phages.
**Table S2.** Head and tail length of phages KN1‐1, KN3‐1, and KN4‐1.
**Table S3.** Primers used in this study.
**Table S4.** *Klebsiella* phages and phage‐borne capsule depolymerases.
