# Supplementary material for: Identification of three podoviruses infecting Klebsiella encoding capsule depolymerases that digest specific capsular types
Source: Microb Biotechnol. 2019 Jan 31;12(3):472–86. doi: 10.1111/1751-7915.13370 (PMC6465236; doi:10.1111/1751-7915.13370)
Supplement: Supplementary file 1 — Fig. S1. Infectivity of phages KN1‐1, KN3‐1, and KN4‐1 toward different strains. Fig. S2. Co‐incubation of phage KN4‐1 with 1461 or 4565. Fig. S3. Alcian blue staining of CPS from K56 reference strain and Δorf8‐orf11 of K56. Fig. S4. Adsorption experiments of phage KN3‐1. Table S1. Klebsiella strains used in this study and the host range of phages. Table S2. Head and tail length of phages KN1‐1, KN3‐1, and KN4‐1. Table S3. Primers used in this study. Table S4. Klebsiella phages and phage‐borne capsule depolymerases. [file MBT2-12-472-s001.docx]

**Table S1. *Klebsiella* strains used in this study and the host range of phages**

| Capsular type | Strain | Species | Note | ФKN1-1 | ФKN3-1 | ФKN4-1 |
| --- | --- | --- | --- | --- | --- | --- |
| K1 | A5054 | *K. pneumoniae* | Reference strain | - | - | - |
|  | NTUH-K2044 | *K. pneumoniae* | (Lin et al., 2014) | - | - | - |
| K2 | B5055 | *K. pneumoniae* | Reference strain | - | - | - |
| K3 | C5046 | *K. pneumoniae* | Reference strain | - | - | - |
| K4 | D5050 | *K. pneumoniae* subsp. *ozaenae* | Reference strain | - | - | - |
| K5 | E5051 | *K. pneumoniae* subsp. *ozaenae* | Reference strain | - | - | - |
| K6 | F052 | *K. pneumoniae* subsp. *ozaenae* | Reference strain | - | - | - |
| K7 | Aerogenes 4140 | *K. pneumoniae* | Reference strain | - | - | - |
| K8 | *Klebsiella* 1015 | *K. pneumoniae* | Reference strain | - | - | - |
| K9 | *Klebsiella* 1056 | *K. pneumoniae* | Reference strain | - | - | - |
| K10 | *Klebsiella* 919 | *K. pneumoniae* | Reference strain | - | - | - |
| K11 | *Klebsiella* 390 | *K. pneumoniae* | Reference strain | - | - | - |
| K12 | *Klebsiella* 313 | *K. pneumoniae* | Reference strain | - | - | - |
| K13 | *Klebsiella* 1470 | *K. pneumoniae* | Reference strain | - | - | - |
| K14 | 138 | *K.* (*Raoultella*) *planticola* | Reference strain | - | - | - |
| K15 | Mich. 61 | *K. pneumoniae* | Reference strain | - | - | - |
| K16 | 2069/49 | *K. pneumoniae* | Reference strain | - | - | - |
| K17 | 2005/49 | *K. pneumoniae* | Reference strain | - | - | - |
| K18 | 1754/49 | *K. pneumoniae* | Reference strain | - | - | - |
| K19 | 293/50 | *K. pneumoniae* | Reference strain | - | - | - |
| K20 | 889/50 | *K. pneumoniae* | Reference strain | - | - | - |
| K21 | 1702/49 | *K. pneumoniae* | Reference strain | - | - | - |
| K22 | 1996/49 | *K. pneumoniae* | Reference strain | - | - | - |
| K23 | 2812/50 | *K. pneumoniae* | Reference strain | - | - | - |
| K24 | 1680/49 | *K. pneumoniae* | Reference strain | - | - | - |
| K25 | 2002/49 | *K. pneumoniae* | Reference strain | - | - | - |
| K26 | 5884 | *K. oxytoca* | Reference strain | - | - | - |
| K27 | 6613 | *K. pneumoniae* | Reference strain | - | - | - |
| K28 | 5758 | *K. pneumoniae* | Reference strain | - | - | - |
| K29 | 5725y | *K. oxytoca* | Reference strain | - | - | - |
| K30 | 7824 | *K. pneumoniae* | Reference strain | - | - | - |
| K31 | 6258 | *K. pneumoniae* | Reference strain | - | - | - |
| K32 | 6837 | *K.* (*Raoultella*) *ornithinolytica* | Reference strain | - | - | - |
| K33 | 6168 | *K. pneumoniae* | Reference strain | - | - | - |
| K34 | 7522 | *K. pneumoniae* | Reference strain | - | - | - |
| K35 | 7444 | *K.* (*Raoultella*) *planticola* | Reference strain | - | - | - |
| K36 | 8306 | *K. pneumoniae* | Reference strain | - | - | - |
| K37 | 8238 | *K. pneumoniae* | Reference strain | - | - | - |
| K38 | 8414 | *K. pneumoniae* | Reference strain | - | - | - |
| K39 | 7749 | *K. pneumoniae* | Reference strain | - | - | - |
| K40 | 8588 | *K. pneumoniae* | Reference strain | - | - | - |
| K41 | 6177 | *K. michiganensis* | Reference strain | - | - | - |
| K42 | 1702 | *K. pneumoniae* | Reference strain | - | - | - |
| K43 | 2482 | *K. pneumoniae* | Reference strain | - | - | - |
| K44 | 7730 | *K.* (*Raoultella*) *ornithinolytica* | Reference strain | - | - | - |
| K45 | 8464 | *K. pneumoniae* | Reference strain | - | - | - |
| K46 | 5281 | *K. pneumoniae* | Reference strain | - | - | - |
| K47 | 9682 | *K. pneumoniae* | Reference strain | - | - | - |
| K48 | 1196 | *K. variicola* | Reference strain | - | - | - |
| K49 | 6115 | *K. variicola* | Reference strain | - | - | - |
| K50 | 1303/50 | *K. pneumoniae* II-B | Reference strain | - | - | - |
| K51 | 4715/50 | *K. pneumoniae* | Reference strain | - | - | - |
| K52 | 5759/50 | *K. pneumoniae* | Reference strain | - | - | - |
| K53 | 1756/51 | *K. variicola* | Reference strain | - | - | - |
| K54 | Stanley | *K. variicola* | Reference strain | - | - | - |
| K55 | 3985/51 | *K. pneumoniae* | Reference strain | - | - | - |
| K56 | 3534/51 | *K. variicola* | Reference strain | - | + | - |
| K57 | 4425/51 | *K. variicola* | Reference strain | - | - | - |
| K58 | 636/52 | *K. variicola* | Reference strain | - | - | - |
| K59 | 2212/52 | *K. michiganensis* | Reference strain | - | - | - |
| K60 | 4463/52 | *K. pneumoniae* II-B | Reference strain | - | - | - |
| K61 | 5710/52 | *K. pneumoniae* | Reference strain | - | - | - |
| K62 | 5711/52 | *K. pneumoniae* | Reference strain | - | - | - |
| K63 | 5845/52 | *K. pneumoniae* | Reference strain | - | - | - |
| K64 | NCTC 8172 | *K. pneumoniae* | Reference strain | - | - | - |
| K65 | SW4 | *K.* (*Raoultella*) *terrigena* | Reference strain | - | - | - |
| K66 | 438(3a) | *K. michiganensis* | Reference strain | - | - | - |
| K67 | 264(1) | *K.* (*Raoultella*) *terrigena* | Reference strain | - | - | - |
| K68 | 265(1) | *K.* (*Raoultella*) *terrigena* | Reference strain | - | - | - |
| K69 | 889 | *K.* (*Raoultella*) *terrigena* | Reference strain | - | - | - |
| K70 | 167 | *K. michiganensis* | Reference strain | - | - | - |
| K71 | 4349 | *K. variicola* | Reference strain | - | - | - |
| K72 | 1205 | *K.* (*Raoultella*) *ornithinolytica* | Reference strain | - | - | - |
| K74 | 371 | *K. oxytoca* | Reference strain | - | - | - |
| K79 | 325 | *K.* (*Raoultella*) *planticola* | Reference strain | - | - | - |
| K80 | 708 | *K. pneumoniae* II-B | Reference strain | - | - | - |
| K81 | 370 | *K. pneumoniae* | Reference strain | - | - | - |
| K82 | 3454-70 | *K. pneumoniae* | Reference strain | - | - | - |
| KN1 | A1517 | *K. pneumoniae* | (Pan et al., 2008) | + | - | - |
|  | 6451N | *K. pneumoniae* | NTUH^a^ | + | - | - |
|  | Ca0514 | *K. pneumoniae* | (Hsu et al., 2013) | + | - | - |
| KN2 | Ca0507 | *K. pneumoniae* | (Hsu et al., 2013) | - | - | - |
| KN3 | N386-KCR59 | *K. pneumoniae* | (Pan et al., 2015) | - | + | - |
|  | N345-2-KCR57 | *K. pneumoniae* | (Pan et al., 2015) | - | + | - |
|  | N348-KCR58 | *K. pneumoniae* | (Pan et al., 2015) | - | + | - |
|  | 1595E | *K. pneumoniae* | NTUH^a^ | - | + | - |
|  | 2283219 | *K. pneumoniae* | NTUH^a^ | - | + | - |
| KN4 | 1461 | *K. pneumoniae* | (Pan et al., 2013) | - | - | + |
|  | 4565 | *K. pneumoniae* | NTUH^a^ | - | - | + |
|  | 4486-2 | *K. pneumoniae* | NTUH^a^ | - | - | + |
|  | 7966E | *K. pneumoniae* | NTUH^a^ | - | - | + |
|  | 2139670 | *K. pneumoniae* | NTUH^a^ | - | - | + |
|  | 3669933 | *K. pneumoniae* | NTUH^a^ | - | - | + |
| KN5 | Ca0431 | *K. pneumoniae* | (Pan et al., 2017)  of Manitoba | - | - | - |

Note: ^a^NTUH, National Taiwan University Hospital

**Table S2. Head and tail length of phage KN1-1, KN3-1, and KN4-1**

|  | KN1-1 | | KN3-1 | | KN4-1 | |
| --- | --- | --- | --- | --- | --- | --- |
|  | Head | Tail | Head | Tail | Head | Tail |
| Average (nm) | 60.05 | 14.5 | 47.85 | 14.98 | 43.24 | 12.95 |
| Relevant SD | 2.59 | 1.52 | 1.63 | 1.39 | 1.65 | 0.44 |
| No. of measurements | 11 | 6 | 6 | 5 | 8 | 3 |

**Table S3. Primers used in this study**

| Primer name | Sequences | Purpose |
| --- | --- | --- |
| 1517 en start 2mF | CCATGATGAACCAAGATATTAAA | KN1 dep expression |
| 1517 en stop-4R | GATAAGGGAACGTAGTCGATTC | KN1 dep expression |
| N56-2 ORF1  Histag for(NheI) | CAACTAAAGGAGGCTAGCATGGACCAAGAC | KN3 dep expression |
| N56-2 ORF1  Histag re (XhoI) | CTTAAGCATGATCTCGAGTTAATATAGTTGACGCC | KN3 dep expression |
| N56-2 ORF2 histag Sac1-F | CAACTATATTAAGGAGCTCTCATGCTTAAGACCG | K56 dep expression |
| N56-3 ORF2 histag re (XhoI) | GGAAACATGTTACCCTCGAGTTATCTGAACCAC | K56 dep expression |
| N3-BamHI(+2)-F | CTTCTCATTCAACTAAAGGGGATCCCAATGGACCAAGAC | KN4 dep expression |
| N3-SacI(+1)-R | GTTGGTTAGGGAAGAGCTCGCCTCCTTACGCTAATGTCAATG | KN4 dep expression |
| K56-WbaP-Ff | GGACTCTGGAGAAGAGATAC | K56 mutant construct |
| K56-ORF12-Rf | GAGATCGTACCACCATATGC | K56 mutant construct |
| K56-WbaP-Ri | GCACAATTGTTATTAATTACGC | K56 mutant construct |
| K56-ORF12-Fi | AATAAGGAGTGAAGAGTG | K56 mutant construct |

Table S4. *Klebsiella* phages and phage-borne capsule depolymerases

| Phage | Phage genome accession no. | Host | Capsule depolymerase | Capsule depolymerase accession no. | Capsule depolymerase activity | Reference |
| --- | --- | --- | --- | --- | --- | --- |
| NTUH-K2044-K1-1 | AB716666 | K1 | K1-ORF34 | N.A. | K1 | (Lin et al., 2014) |
| K64-1 | LC121097 | K1, K11, K21, K25, K30, K35, K64, K69, KN4, KN5 | S1-1 | YP_002003830.1 | K11 | (Pan et al., 2017) |
|  |  |  | S1-2 | YP_008532048.1 | KN4 |  |
|  |  |  | S1-3 | AGF88658.1 | K21 |  |
|  |  |  | S2-1 | YP_007010682.1 | KN5 |  |
|  |  |  | S2-2 | WP_020326882.1 | K25 |  |
|  |  |  | S2-3 | YP_654147.1 | K35 |  |
|  |  |  | S2-4 | YP_007003187.1 | K1 |  |
|  |  |  | S2-5 | YP_398994.1 | K64 |  |
|  |  |  | S2-6 | WP_020801644.1 | K30/K69 |  |
| KpV71 | KU666550 | K1, K62 | kpv71_52 | AMQ66478 | K1 | (Solovieva et al., 2018) |
| KpV74 | KY385423 | K2, K13 | kpv74_56 | APZ82768.1 | K2/K13 | (Solovieva et al., 2018) |
| K5-2 | KY389315 | K5, K30, K69 | K5-2 ORF37 | N.A. | K30/K69 | (Hsieh et al., 2017) |
| K5-4 | KY389316 | K5, K8 | K5-4 ORF37 | N.A. | K8 | (Hsieh et al., 2017) |
|  |  |  | K5-4 ORF38 | N.A. | K5 |  |
| KP36 | NC_029099 | K63 | depoKP36 | YP_009226010.1 | K63 | (Majkowska-Skrobek et al., 2016) |
| 0507-KN2-1 | AB797215 | KN2 | ORF96 | N.A. | KN2 | (Hsu et al., 2013) |
| KP32 | NC_013647 | K3, K21 | KP32gp37 | N.A. | K3 | (Majkowska-Skrobek et al., 2018) |
|  |  |  | KP32gp38 | N.A. | K21 |  |

N.A., not available


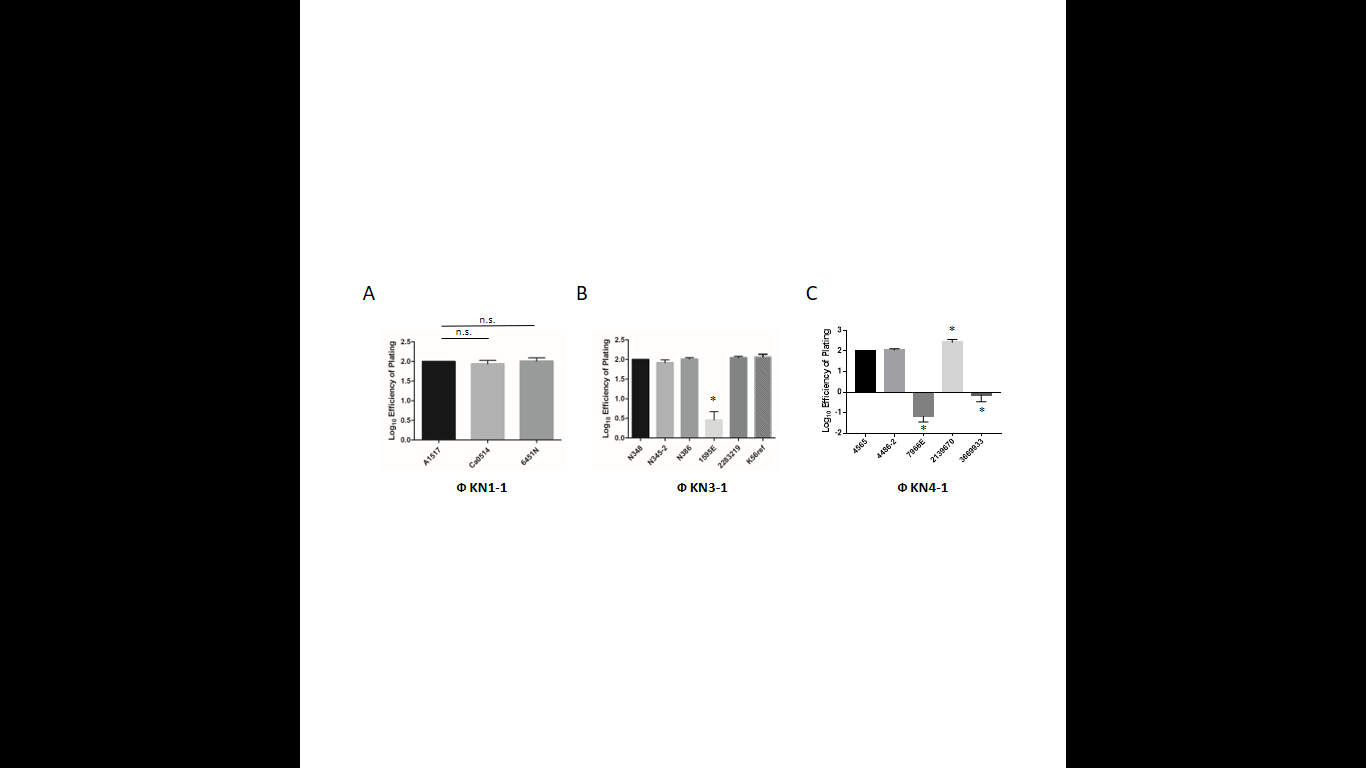


**Fig S1. Infectivity of** **phages KN1-1, KN3-1, and KN4-1 toward different strains.**

An efficiency-of-plating (EOP) assay was used to quantitate the ability of phage to infect different hosts. The original titers of phage KN1-1, KN3-1, and KN4-1 were determined using A1517, N348, and 4565, respectively. The EOP of phages against A1517, N348, and 4565 were set to 100(%) for each group and the ratio of other strain to the host used for original titer determination was calculated. For example, the ratio of Ca0514 was calculated by the phage titer on Ca0514 compared to the titer on A1517. The EOP (Log_10_) was shown from three independent experiments (mean ± standard deviation [SD]). *P < 0.05 by paired *t* test; n.s., not significant (P > 0.05).

*A*, phage KN1-1 and KN1 hosts (A1517, Ca0514, and 6451N); *B*, phage KN3-1 and KN3 hosts (N348, N345-2, N386, 1595E, and 2283219), and K56 reference strain (abbreviated as K56ref); *C*, phage KN4-1 and KN4 hosts (4565, 4486-2, 7966E, 2139670, and 3669933).

**
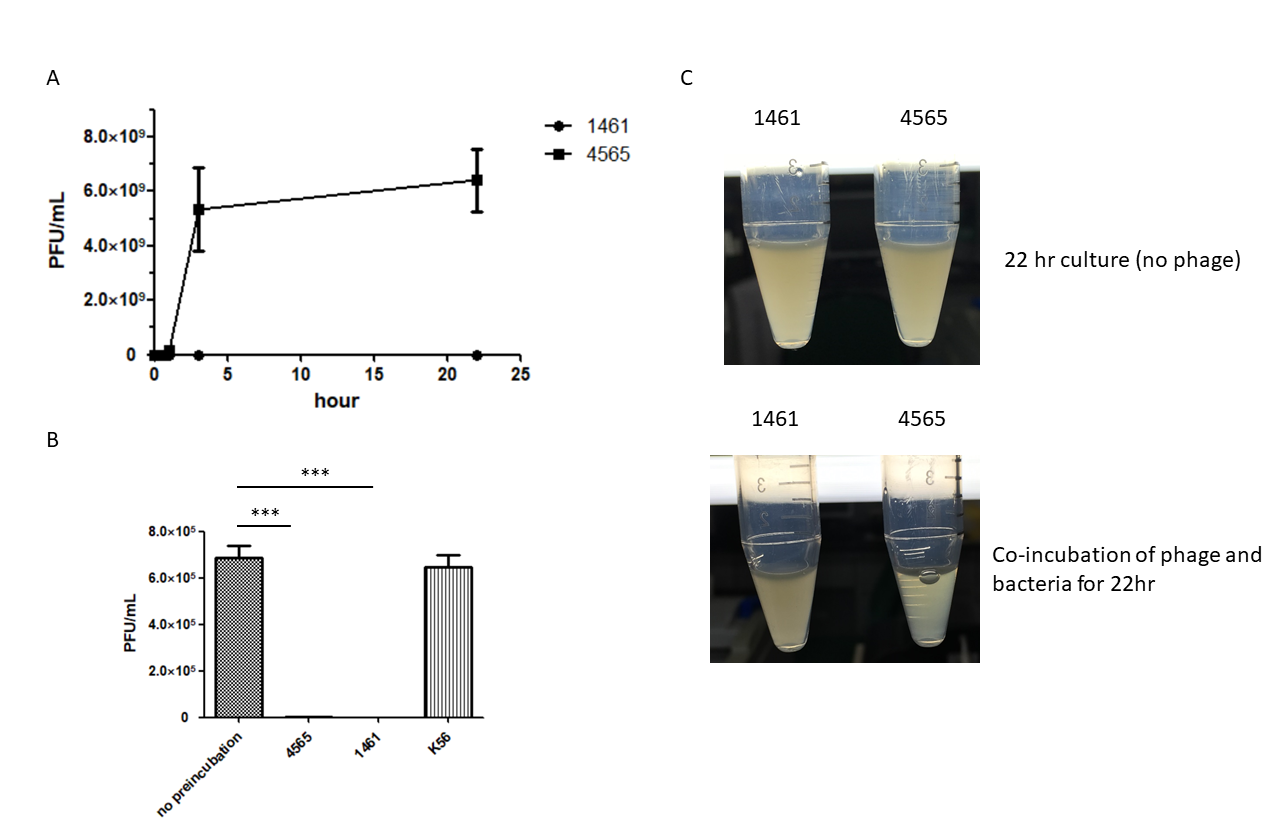
**

**Fig S2. Co-incubation of phage KN4-1 with 1461 or 4565**

**(A) phage propagation.** A total of 5×10^3^ pfu of phage KN4-1 was mixed with 5×10^6^ cfu of 1461 or 4565 strain individually and incubated at 37 °C. Samples were taken at 0.5, 1, 3, and 22 hours. For plaque counting, 100 μl of phage suspension was mixed with 100 μl of exponential phase culture of 4565 strain (we use 4565 but not 1461 because phage numbers can be estimated by calculating the visible plaques on 4565) for 10 minutes, mixed with top agar, and poured on a LB agar. **(B) Phage adsorption assay.** A phage preparation of 1×10^6^ pfu was made for phage KN4-1 and the titer was determined using 4565 strain. Adsorption assay was performed by preincubating phage KN4-1 with 1×10^9^ cfu of 4565, 1461, and K56 (as a control) in a total of 1 ml individually. After preincubation for 5 minutes at room temperature, the mixture was immediately filtered by 0.45 µm pore size hydrophilic Polyethersulfone (PES) membrane. Phage particles that had attached to bacteria will be eliminated from the filtrate (the remaining would be unadsorpted phages). The filtrate was then titered on 4565. ***P < 0.001 by Student’s t test. (C) After 22 hr incubation with 4565, the bacterial culture appeared clear (lysis), in contrast, bacterial culture of 1461 showed turbid (growth).


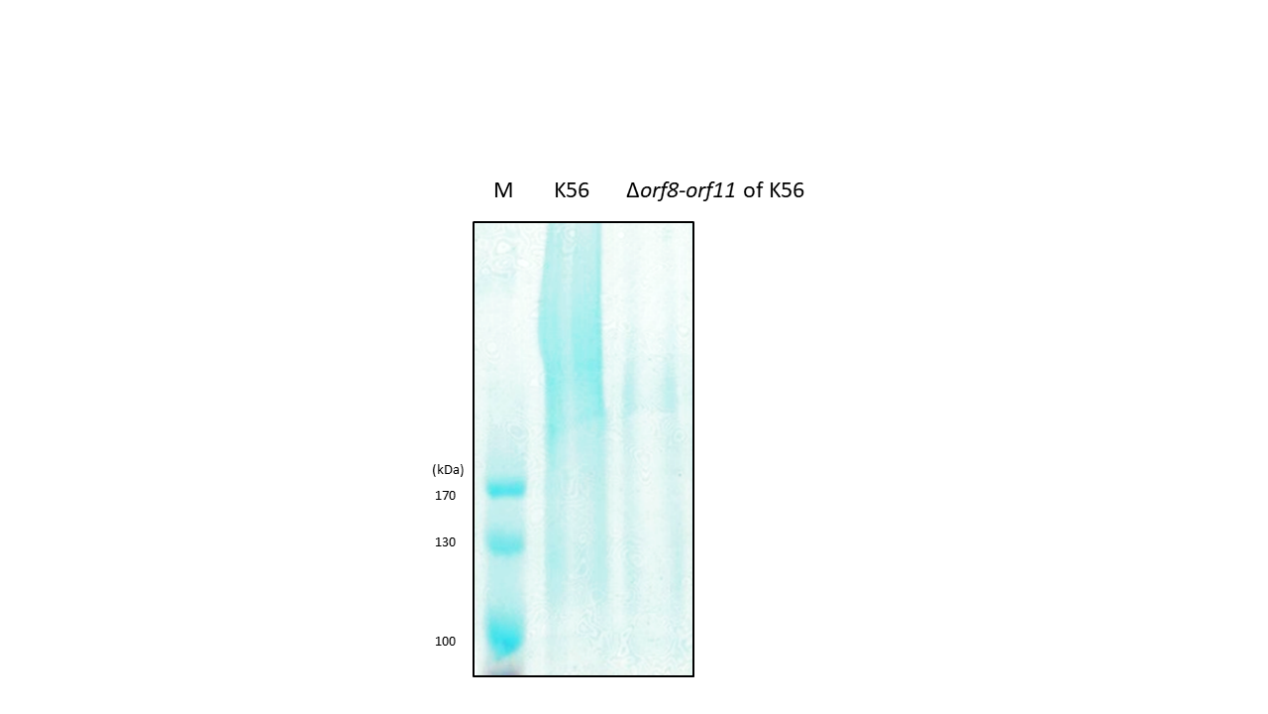


**Figure S3. Alcian blue staining of CPS from K56 reference strain and Δ*orf8-orf11*** of K56. Extracellular polysaccharides were extracted from 1×10^8^ cfu of K56 reference strain and Δ*orf8-orf11* of K56 and then visualized by Alcian blue staining. Lane 1 (M) is a protein marker; lane 2 is K56 reference strain; lane 3 is Δ*orf8-orf11* of K56.


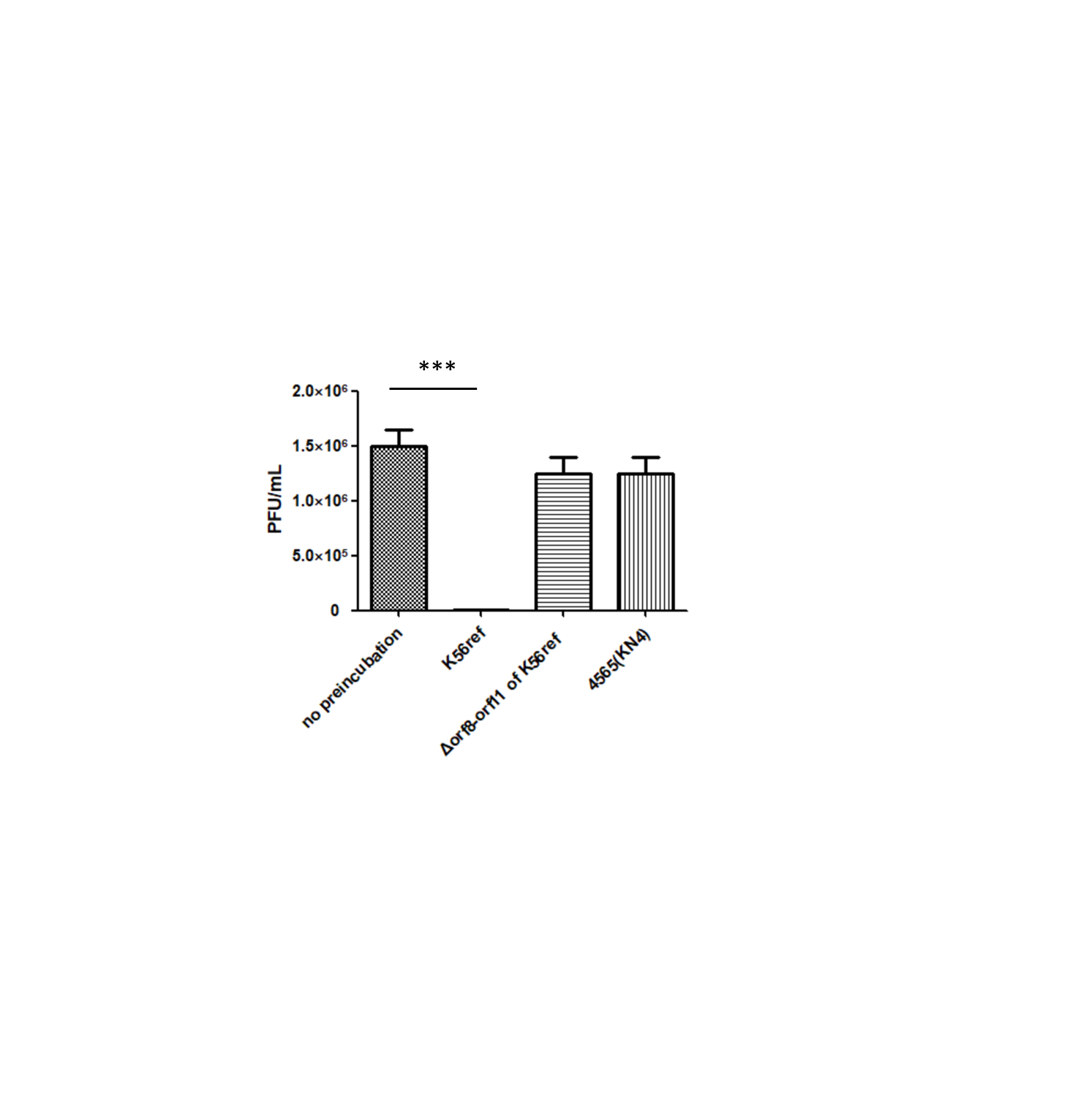


**Figure S4. Adsorption experiments of phage KN3-1.**

Preincubation of phage KN3-1 with K56 reference strain, Δ*orf8-orf11* of K56 or a non-KN3-1 host (4565- KN4 strain) and determination of the unadsorpted viral titers on K56 strain. The data represent the means of three independent trials; the error bars represent the standard deviations. ***P < 0.001 by Student’s t test. K56 reference strain is abbreviated as K56ref.
